# Acanthamoeba keratitis challenges a case report

**Published:** 2016

**Authors:** Stan Cristina, Vlăduţiu Cristina, Popovici Mihaela

**Affiliations:** * Ophthalmology Clinic, County Clinical Emergency Hospital of Cluj-Napoca, Cluj, Romania; ** ”Iuliu Haţieganu” University of Medicine and Pharmacy, Cluj-Napoca, Cluj, Romania

**Keywords:** Acanthamoeba keratitis, discoid opacity, corneal infiltrate

## Abstract

Acanthamoeba keratitis is a rare, chronic, mainly contact lens-related infection caused by a free-living amoeba found ubiquitously in water and soil.

A case of a 9-year-old child, who presented to our clinic with painful, red left eye, associated with photophobia, and decreased visual acuity, wais reported. The clinical examination revealed a discoid opacity inferiorly bounded by a dense, gray infiltrate. The progressive nature of the corneal infiltrate, the epithelial defect, and the lack of response to treatment was highly suggestive for Acanthamoeba keratitis.

The distinctiveness of this case was the presence of Acanthamoeba keratitis in a child without a history of trauma or contact lens usage, the lack of an appropriate diagnosis and management of this vision-threatening infection.

## Introduction

Acanthamoeba keratitis is a sight- threatening infection caused by a free-living, pathogenic amoeba. It causes a progressive ulcerative keratitis, which is not replying to the common antimicrobial therapy and is frequently misdiagnosed for stromal herpes keratitis [**1**].

Acanthamoeba is naturally found in air, soil, and water and is relatively resistant to normal levels of chlorine in tap water [**2**]. It exists in two forms: as an invasive, trophozoite stage and as a latent, cystic stage [**3**].

The earliest evidence of acanthamoeba infection is the diffuse, irregular edema, which occurs at the epithelial level and may lead to a dendritiform ulceration, which is often mistakenfor herpes simplex virus keratitis [**1**]. There are certain clinical features that may prompt Acanthamoeba identification. Anterior stromal infiltration as a partial or complete circle tends to be paracentral with clear central cornea in early disease. Unbearable pain is pathognomonic and is usually due to perineural infiltration. If left untreated, the amoeba invades all layers of the cornea, determining a ring abscess, which may ultimately end with a corneal perforation [**3**].

Diagnosis is made upon the direct visualization of the Acanthamoeba by confocal microscopy. Cysts appear as round, double- walled and hyper-reflective structures. Besides, cysts could be visible with regular Giemsa, Gram’s, ink-potassium hydroxide stains or the ones that require fluorescent microscopy such as acridine orange and Calcofluor white. Commonly, Acanthamoeba can be grown on a bed of E. Coli plated on a non-nutrient agar. Other investigations include PCR or corneal biopsy [**4**].

The typical treatment consists of hourly, around-the-clock, topical applications of Biguanide (polyhexamethylene biguanide – PHMB 0.02% and chlorhexidine - CHX 0.02%), and diamide (Brolene 0.1% or hexamidine), alone or in combination. Debridement of affected epithelium may aid eye drop penetration. Antifungals such as Voriconazole or other azoles may be efficient. Antibacterial treatment for co-infection may be advised if the clinical picture encourages it. Steroid therapy (oral or topical) may help control the inflammation after the control of the infection has been attained. Penetrating Keratoplasty (PKP) may be necessary for resistant cases, for poor visual acuity after scarring or imminent perforation [**5**].

## Case report

A 9 year-old child presented to our clinic with painful, red left eye, photophobia, tearing and decreased visual acuity. Three weeks prior to the presentation to our clinic, the patient began to develop cloudy vision, photophobia, and increasingly exquisite pain in the left eye. The symptoms worsened despite topical antibiotic therapy. Before the arrival to our clinic, the patient had been unsuccessfully treated with acyclovir, ibuprofen, topical antibiotic, and mydriatic for presumed herpes simplex virus keratitis. The medical history highlighted a possible corneal abrasion due to intense scratching and the use of tap water to wash the eyes.

**Fig. 1:**
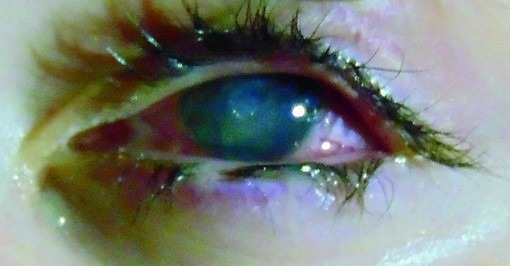
Left eye ciliary injection, central discoid opacity with epithelial defect

The best-corrected visual acuity was 5/ 5 in the right eye and hand-motion vision in the left eye. A right eye slit lamp examination was normal, while a left eye slit lamp examination showed a marked ciliary injection and diffuse corneal edema. The central part of the cornea was involved with a discoid opacity bounded inferiorly by a dense, gray infiltrate, which made the examination of the anterior chamber impossible. There was an epithelial defect overlying that area and endothelial precipitates under the infiltrate. The pupil was miotic (**Fig. 1**).

According to this picture, the treatment with local mydriatics, corneal reepithelialization agents, and artificial tears was initiated. The patient received various antimicrobial medications, including topical ciprofloxacin 0.3%, moxifloxacine 0.5%, fluconazole 0.2%, acyclovir ointment and systemic ceftriaxone, cefuroxime, gentamicin, acyclovir. Intravenous dexamethasone (8mg/ day-> 4mg/ 2days->4mg/ 3 days) and a nonsteroidal inflammatory drug (ibuprofen) were administered. A therapeutic bandage contact lens was applied to relieve the pain but it was borne away due to the distress and purulent secretion. The patient received autologous platelet-rich plasma eye drops along with standard medical treatment. Direct bacteriological examination of conjunctival secretion was negative for bacteria or fungi.

Along hospitalization, the evolution was fluctuating. The stromal infiltrate worsened and a translucent crescent formed at the edge of the infiltrate with deep stromal neovascularization. The Acanthamoeba keratitis was highly suspected due to the progressive nature of the corneal infiltrate, the epithelial defect, and the lack of response to treatment.

A confocal microscopy examination was performed in the Ophthalmology Department of University of Debrecen and revealed characteristic cyst-like structures in and on the surface of the corneal infiltrate. Acanthamoeba keratitis was confirmed.

Since the standard treatment is currently unavailable in Romania, the parents transported the patient to an ophthalmology clinic in Belgium where she received the appropriate care.

## Discussions

The nonspecific clinical features of early stages and the diverse morphological manifestations could often postpone Acanthamoeba keratitis diagnosis. Due to deep inflammation and persistent epithelial defects, the infection is often confounded with herpes simplex stromal keratitis [**4**]. The clinical aspect of the ulceration and the progressive nature of the corneal infiltrate were highly suggestive for Acanthamoeba keratitis.

Intense ocular pain due to infection or inflammatory process should signal Acanthamoeba keratitis [**1**]. Despite the anti- inflammatory agents, the disapproval of the therapeutic bandage contact lens and the sharp ocular pain emphasizes this evidence.

In cases of keratitis in children, acanthamoeba should be regarded even without the history of contact lens usage [**6**]. Acanthamoeba infection was most probably the consequence of the intense eye scratching during a keratitis episode and the contamination from tap water the parents reported.

This case was a true challenge for several reasons. The management of keratitis was particularly complicated by the poor cooperation during the examinations and the lack of information prior to the presentation. Moreover, the lack of appropriate diagnosis tools and medical therapy in Romania, led to a failure regarding the diagnosis and management of this sight threatening infection, resistant to most ocular antibiotics.
